# Exploring transdiagnostic factors for mental health screening in primary care: a secondary analysis of a randomised controlled pilot study

**DOI:** 10.1186/s13104-026-07885-5

**Published:** 2026-05-29

**Authors:** Christopher Ebert, Óscar Peris-Baquero, Lukas Junker, Thomas Ehring, Jochen Gensichen, Jorge Osma

**Affiliations:** 1 Institute of General Practice and Family Medicine, University Hospital, LMU Munich, Munich, Germany; 2 DZPG (German Center for Mental Health) , Partner Site Munich/Augsburg, Germany; 3 Department of Psychology and Sociology, Universidad de Zaragoza, Teruel, Spain; 4 Instituto de Investigación Sanitaria de Aragón, Zaragoza, Spain; 5 Department of Psychology, LMU Munich, Munich, Germany

**Keywords:** Primary care, Family medicine, General practitioner, Transdiagnostic, Unified protocol, Brief psychological intervention, Psychoeducation, Mental health, Pilot study, Randomised controlled trial

## Abstract

**Objective:**

This secondary analysis of a randomised controlled pilot study (*n* = 87 patients) examined how transdiagnostic factors-including emotion regulation mechanisms (emotion beliefs, cognitive reappraisal, emotion suppression and experiential avoidance) and negative affectivity-relate to depressive, anxiety and somatic symptoms in the primary care setting, and whether emotion regulation mechanisms provide potential screening relevance beyond negative affectivity, i.e., by explaining variance in symptom severity that is not accounted for by negative affectivity alone.

**Results:**

All transdiagnostic factors were correlated with depressive, anxiety and somatic symptom scores. Negative affectivity showed the strongest associations across diagnosis-specific symptom scores, while small to moderate correlation estimates were observed for the emotion regulation mechanisms. In multiple regression analyses, negative affectivity remained the most consistent contributor to symptom variance; emotion regulation mechanisms showed limited incremental value. Lastly, across the respective symptom severity levels of depression, anxiety and somatic symptom scores, negative affectivity most closely reflected symptom gradients. These cross-sectional findings suggest that negative affectivity may serve as a particularly informative transdiagnostic indicator of overall symptom burden in primary care. While no incremental relevance of emotion-regulation mechanisms beyond negative affectivity was supported, their potential for guiding mechanism-based interventions and relation to symptom trajectories should be examined by future research.

***Trial registration:*** The pilot study has been registered with the German Clinical Trials Register: 18^th^ of March 2024, https://drks.de/search/en/trial/DRKS00033386

**Supplementary Information:**

The online version contains supplementary material available at10.1186/s13104-026-07885-5.

## Introduction

Primary care (PC) is a central point of contact for individuals experiencing mental health symptoms [1] and typically represents the first step before accessing specialised mental healthcare [2]. General practitioners (GPs) therefore require screening tools that support the accurate identification of mental health conditions [3]. Unlike the psychiatric setting, PC involves no prior diagnostic pre-selection [4]. Patients often present with diverse, vague or overlapping symptom profiles that do not map neatly onto single diagnostic categories [5]. This limits the usefulness of diagnosis-specific screening tools [1, 6], which are designed to detect discrete disorders and may fail to account for comorbid or subthreshold symptom presentations [7]. Furthermore, applying several diagnosis-specific measures to find the most fitting diagnostic label is unfeasible within the time constraints of PC consultations [8]. Consequently, broader and conceptually coherent screening approaches may better reflect the clinical reality of PC [9], for which a transdiagnostic perspective [10] offers a promising framework.

Neulinger et al. [11] systematically reviewed transdiagnostic screening tools for PC and identified a small number of available instruments (*n* = 7), noting substantial conceptual heterogeneity. Most screening tools assessed psychopathology by focusing on the mood or anxiety spectrum. To capture unifying and clinically meaningful constructs of psychopathology, the authors highlighted the potential of approaches grounded in maladaptive personality traits (e.g., negative affectivity [12]) or other overarching transdiagnostic factors.

In line with this, the Modified Brief Personality Inventory for DSM-5 (PID5BF+M [13]), has recently been validated in a German PC population. Among the maladaptive personality domains, negative affectivity (i.e., neuroticism) showed the strongest correlations with depressive, anxiety and somatic symptoms. Thus, it was suggested as a clinically relevant marker for common mental health conditions in PC [14]. More specifically, its clinical relevance lies in capturing an individual’s general propensity to experience intense negative emotions and to engage in maladaptive coping strategies [15], both of which characterise a broad range of internalising symptom presentations [16].

Importantly, however, heightened negative affectivity does not in itself constitute clinical caseness but rather reflects a higher-order vulnerability that is functionally related to lower-order symptom expressions [17]. Transdiagnostic emotion regulation mechanisms have been proposed as processes bridging this conceptual gap [7, 17]. Whereas negative affectivity primarily captures a general tendency to experience negative emotions, emotion regulation mechanisms describe how individuals perceive, process and respond to these emotional experiences [15]. As such, transdiagnostic factors including beliefs about emotions, cognitive reappraisal, emotion suppression and experiential avoidance represent more proximal and potentially modifiable mechanisms [17] and may therefore constitute additional meaningful screening targets in PC.

Using cross-sectional baseline data from a randomised controlled pilot study evaluating a GP-led transdiagnostic intervention [18], the present secondary analysis examined the relationship between transdiagnostic factors (beliefs about emotions, cognitive reappraisal, emotion suppression, experiential avoidance and negative affectivity) and diagnosis-specific symptoms (depressive, anxiety and somatic symptoms). The analysis aimed (1) to quantify the correlations between transdiagnostic and diagnosis-specific measures, (2) to explore how much variance in diagnosis-specific symptom scores is accounted for by the transdiagnostic factors, with particular attention to the potential incremental relevance of emotion regulation mechanisms beyond negative affectivity and (3) to describe the distribution of transdiagnostic factor scores across the respective depressive, anxiety and somatic symptom severity levels.

## Main text

### Methods

#### Study design, participants and procedures

This secondary analysis is based on a cluster-randomised controlled pilot study conducted between April and June 2025 in Munich, Germany [18]. The pilot study evaluated the feasibility, acceptability and potential effectiveness of a GP-led transdiagnostic mental health intervention in PC.

Practices were recruited via the teaching practice network of the Institute of General Practice at LMU Munich. Participating PC practices were randomised (1:1 allocation ratio) to either the intervention group (IG), administering a transdiagnostic intervention, or the control group, providing improved treatment as usual. GPs in both groups recruited adult patients presenting with psychological distress during routine consultations. Identification of eligible patients was based on the GP’s clinical impression, supported by the Kessler Psychological Distress Scale (K-6, [19]). Patients were eligible if they were ≥ 18 years, fluent in German, screened positive for psychological distress and provided written or digital informed consent. Exclusion criteria included severe psychiatric conditions requiring specialised care, acute suicidality, substance use disorder, cognitive impairment, concurrent psychotherapy, recent changes in psychotropic medication, limited life expectancy or inability to attend in-person sessions.

The transdiagnostic intervention in the IG was adapted from the Unified Protocol for Transdiagnostic Treatment of Emotional Disorders (UP), a CBT based psychological intervention focusing on enhancing emotion regulation skills [20]. Treatment comprised four 20-min sessions: session 1 promoted a better understanding of emotions; session 2 addressed cognitive flexibility; session 3 focused on emotion-based avoidance; session 4 summarised treatment content and discussed further care options. In the CG, improved treatment as usual followed official clinical guidelines for depression, anxiety and somatic symptom disorders [21–23]. Apart from four 20-min consultations, no additional structure was required for the CG.

Data were collected before treatment initiation (t_0_; patients only) and after treatment completion (t_1_; patients and GPs), of which only data from t_0_ was used in for this secondary analysis. The study complied with the Declaration of Helsinki [24] and the CONSORT extension for randomised pilot and feasibility studies [25]. Ethical approval was granted by the LMU Munich Ethics Committee (1st of March 2024; ref. no. 24-0080). More detailed information on eligibility criteria, recruitment, randomisation, blinding and treatment content can be derived from the study protocol [26].

#### Outcomes

In the pilot study, feasibility and acceptability were assessed as primary outcomes according to a framework for process evaluations of cluster-randomised controlled trials [27] and procedures from a previous pilot study [28].

Secondary outcomes comprised transdiagnostic factors and diagnosis-specific symptoms, completed by patients pre- and post-treatment. As surrogate measures of potential effectiveness, transdiagnostic factors included maladaptive emotion beliefs [Emotion Beliefs Questionnaire (EBQ [29]); Cronbach’s *α* = 0.87], cognitive reappraisal/emotion suppression [Emotion Regulation Questionnaire (ERQ [30]); Cronbach’s *α* of cognitive reappraisal subscale = 0.84, Cronbach’s *α* of emotion suppression subscale = 0.73], experiential avoidance [Brief Experiential Avoidance Questionnaire (BEAQ [31]; Cronbach’s *α* = 0.92] and negative affectivity [subscale of the Modified Personality Inventory for DSM-5 Brief Form (PID5BF+M [13]); Cronbach’s *α* = 0.74]. Main diagnosis-specific symptom scores of interest were: depressive symptoms [Patient Health Questionnaire-9 (PHQ-9 [32]); Cronbach’s *α* = 0.95], anxiety symptoms [Generalised Anxiety Disorder Screener-7 (GAD-7 [33]); Cronbach’s *α* = 0.87] and somatic symptoms [Patient Health Questionnaire-15 (PHQ-15 [34]); Cronbach’s *α* = 0.60]. In accordance with respective scoring guidelines [32–34], PHQ-9 severity levels were divided into mild (≥ 5), moderate (≥ 10), moderately severe (≥ 15) and severe (≥ 20); for the GAD-7 and PHQ-15, severity was categorised as mild/low (≥ 5), moderate/medium (≥ 10) and severe/high (≥ 15). The study protocol provides an in-depth description of primary and secondary outcomes as well as of additionally collected outcomes (e.g., sociodemographic data) [26].

#### Statistical methods

##### Data analysis

Data analysis was performed with the software R [35]. Cross-sectional baseline data from the IG and CG were analysed jointly. Pearson correlations were used to examine the bivariate associations between diagnosis-specific symptom scores (PHQ-9, GAD-7, PHQ-15) and transdiagnostic factor scores (EBQ, ERQ, BEAQ, PID5BF+M). Associations were additionally illustrated using simple linear regression plots. To further explore the incremental contribution of single transdiagnostic factor scores on diagnosis-specific symptom scores when considered together, separate standardised multiple linear regression models were estimated for each diagnosis-specific symptom score (dependent variable), with all transdiagnostic factors (independent variables) entered simultaneously. To account for minor violations of homoscedasticity observed in the regression diagnostics, robust standard errors (HC3 [36]) were computed. The distribution of transdiagnostic factor scores across the respective depressive, anxiety and somatic symptom severity levels were visualised with scatter plots, including medians and inter-quartile ranges. Missing data (< 1%) were handled using mean imputation. In line with the exploratory nature of the study, estimates are reported with 95% confidence intervals (CIs), without relying on p-values. Accordingly, interpretation focused on the magnitude and direction of effects, with particular attention to estimates whose CIs did not include zero.

##### Sample size

As statistical inference is not the primary aim of a pilot study [25], no a priori sample size calculation was performed. Based on available staff and time resources, and in line with recommendations for minimising the maximum likely error for any estimated rates in pilot studies [37], a recruitment target of 100 patients was set.

### Results

The final sample comprised 87 patients (IG: *n* = 52; CG: *n* = 35). The mean age was 46.98 years (*SD* = 15.21), and 68% of participants were female. Most participants were of German nationality (91%) and over half were married or in a relationship (55%). Educational attainment was generally high, with 49% holding a high school diploma and 33% having a university degree. The majority of participants were employed (83%). At baseline, moderate symptom severity was observed for depressive symptoms (PHQ-9: M = 13.79, *SD* = 5.75), anxiety symptoms (GAD-7: M = 11.89, *SD* = 5.10) and somatic symptoms (PHQ-15: M = 12.78, *SD* = 5.45). One third of participants reported a prior diagnosis of depression (33%), while fewer reported anxiety (8%) or somatic symptom disorders (1%). Further details on baseline characteristics and participant flow have been reported elsewhere [18].

#### Correlations between transdiagnostic factor scores and diagnosis-specific symptom scores

**Fig. 1:**
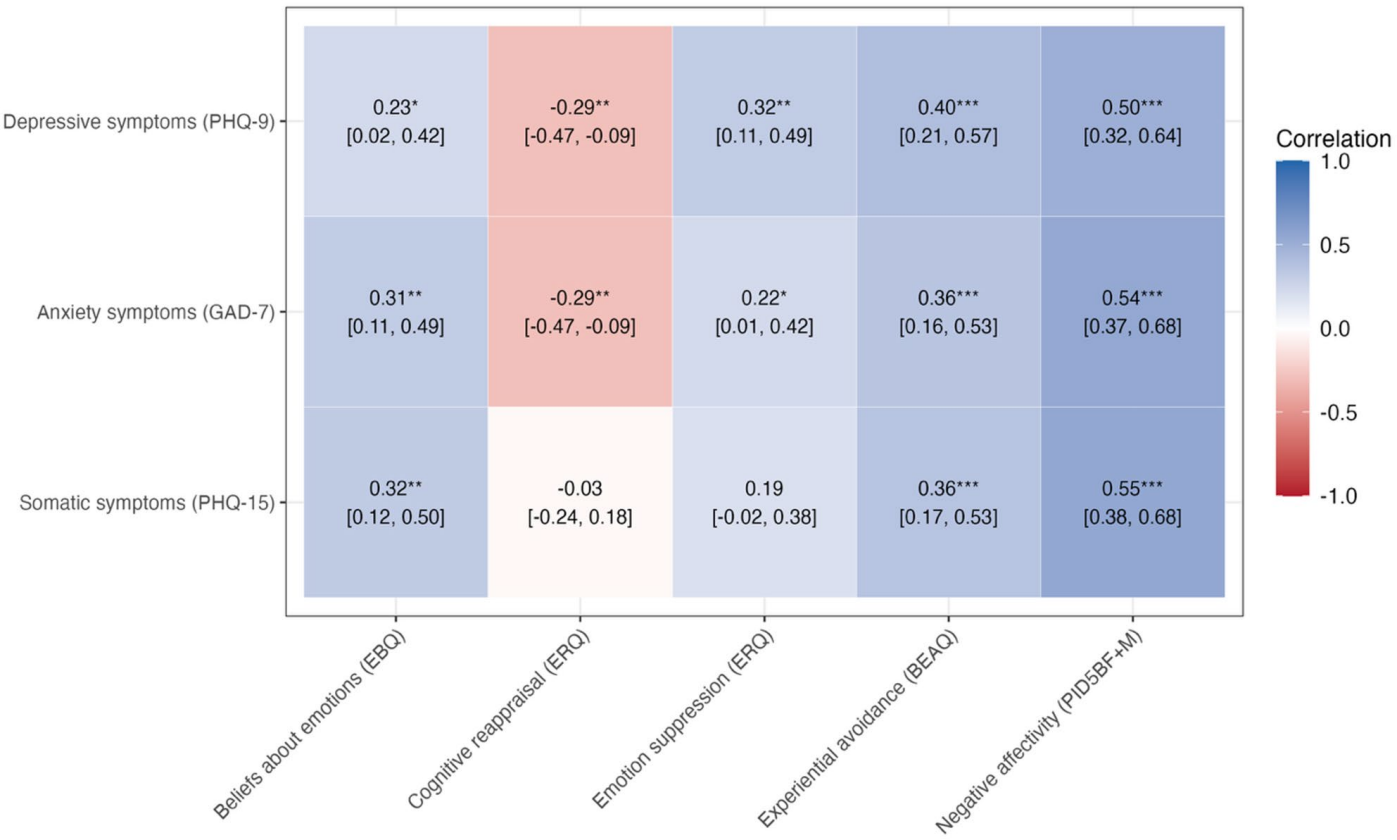
Correlation estimates between transdiagnostic factor scores (EBQ, ERQ, BEAQ, PID5BF + M) and diagnosis-specific symptom scores (PHQ9, GAD7, PHQ15) **Legend:** * = *p* < 0.05; ** = *p* < 0.01; *** = *p* < 0.001; *PHQ-9* Patient Health Questionnaire-9, *GAD-7* Generalised Anxiety Disorder Screener-7, *PHQ-15* Patient Health Questionnaire-15, *EBQ* Emotion Beliefs Questionnaire, *ERQ* Emotion Regulation Questionnaire, *BEAQ* Brief Experiential Avoidance Questionnaire, *PID5BF+M* Modified Personality Inventory for DSM-5 Brief Form

Negative affectivity showed the strongest correlation estimates with depressive, anxiety and somatic symptom scores (PHQ-9: *r* = 0.50, 95% CI [0.32, 0.64]; GAD-7: *r* = 0.54, 95% CI [0.37, 0.68]; PHQ-15: *r* = 0.55, 95% CI [0.38, 0.68]). Among emotion regulation mechanisms, the strongest associations across symptom domains were found for experiential avoidance (PHQ-9: *r* = 0.40, 95% CI [0.21, 0.57]; GAD-7: *r* = 0.36, 95% CI [0.16, 0.53]; PHQ-15: *r* = 0.36, 95% CI [0.17, 0.53]) (Fig. 1). Additional files 1, 2 and 3 present the graphical outputs of single linear regression models for each transdiagnostic factor score in relation to depressive, anxiety and somatic symptom scores, respectively.

#### Multiple linear regression of transdiagnostic factor scores on diagnosis-specific symptom scores

**Fig. 2:**
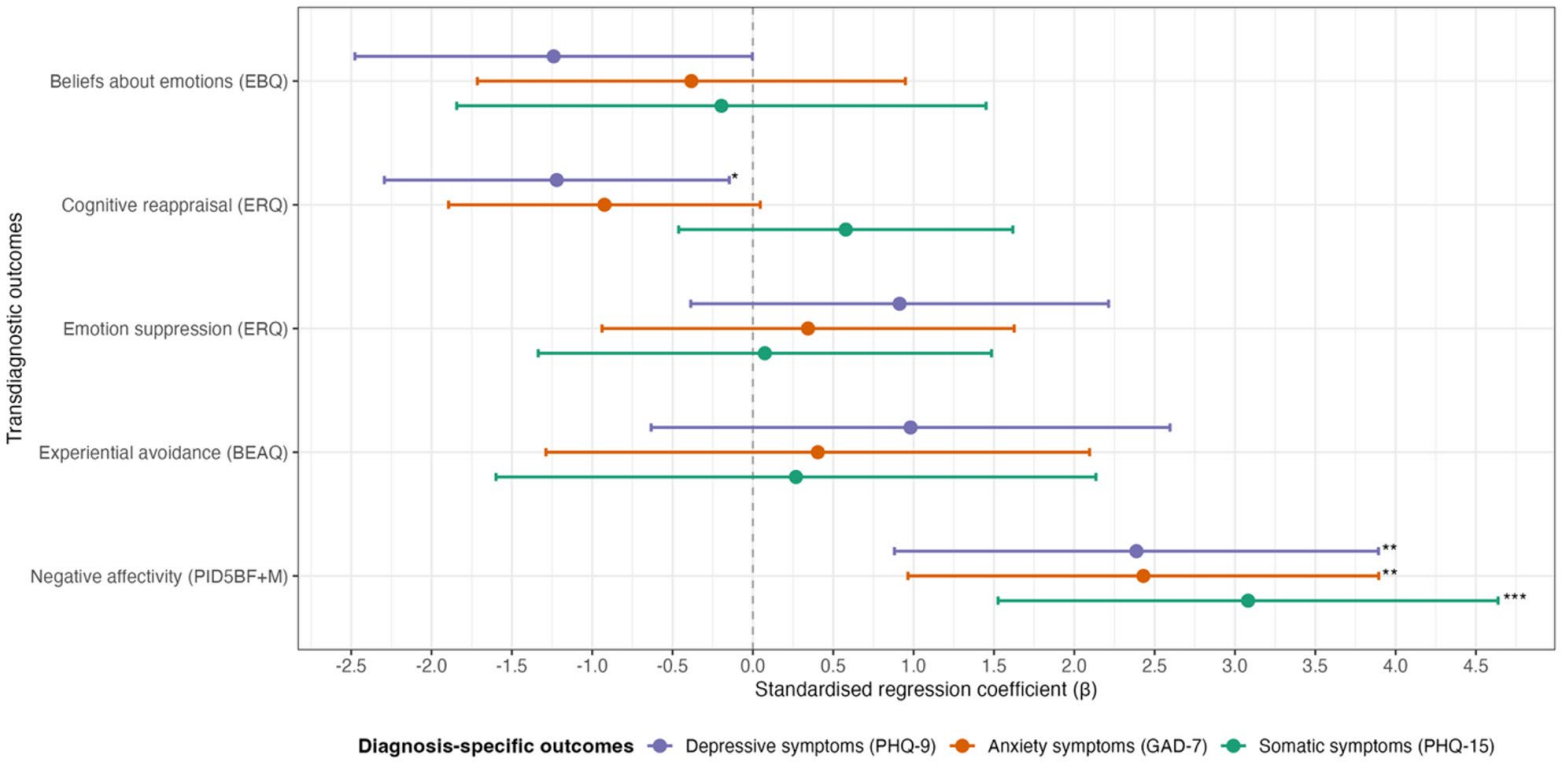
Standardised regression coefficients for associations between transdiagnostic factor scores (EBQ, ERQ, BEAQ, PID5BF + M) and diagnosis-specific symptom scores (PHQ-9, GAD-7, PHQ-15) **Legend:** * = *p* < 0.05; ** = *p* < 0.01; *** = *p* < 0.001; *PHQ-9* Patient Health Questionnaire-9, *GAD-7* Generalised Anxiety Disorder Screener-7, *PHQ-15* Patient Health Questionnaire-15, *EBQ* Emotion Beliefs Questionnaire, *ERQ* Emotion Regulation Questionnaire, *BEAQ* Brief Experiential Avoidance Questionnaire, *PID5BF+M* Modified Personality Inventory for DSM-5 Brief Form

Negative affectivity showed the strongest contribution to explaining variance in depressive, anxiety and somatic symptom scores. Adjusting for all other transdiagnostic factors, an increase in negative affectivity was associated with higher predicted depressive (*ß*: 2.387, 95% CI [0.881, 3.893]), anxiety (*ß*: 2.430, 95% CI [0.956, 3.895]) and somatic symptom scores (*ß*: 3.082, 95% CI [1.526, 4.638]). Out of the different emotion regulation mechanisms, only cognitive reappraisal showed some additional indication of an association with depressive symptoms: higher reappraisal (i.e. lower scores) was linked to lower predicted depression (*ß*: − 1.221, 95% CI [–2.294, –0.147]). Notably, all point estimates for emotion beliefs were negative, associating lower symptom scores with more maladaptive beliefs; though CIs overlapped zero (Fig. 2). Full regression estimates, including R² values and variance inflation factors, are provided in Additional file 4.

#### Distribution of transdiagnostic factor scores across diagnosis-specific symptom severity levels

**Fig. 3:**
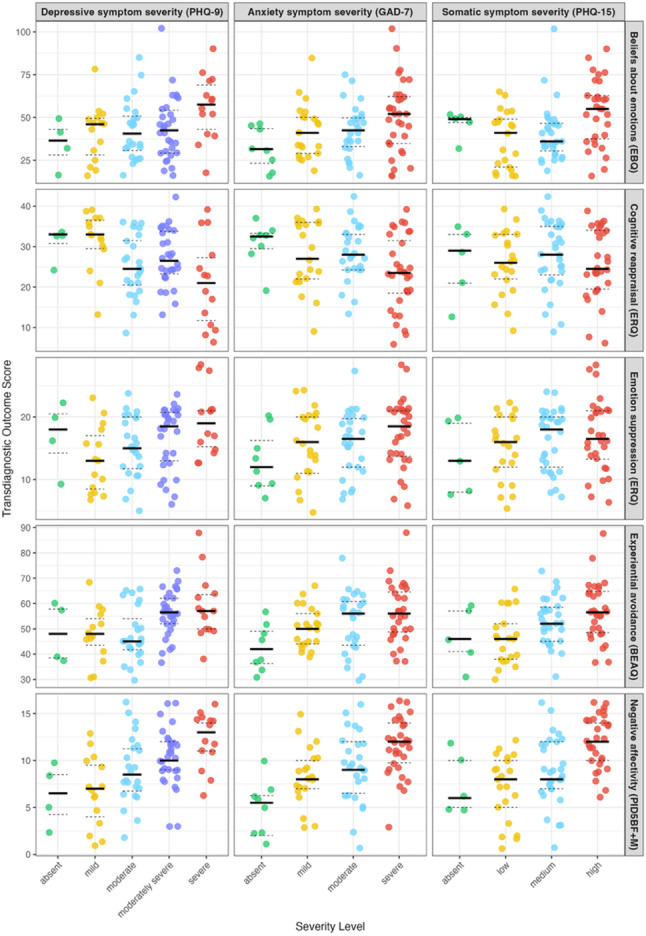
Differences in transdiagnostic factor scores (EBQ, ERQ, BEAQ, PID5BF + M) across severity levels of diagnosis-specific symptom scores (PHQ9, GAD7, PHQ15) **Legend:** *PHQ-9* Patient Health Questionnaire-9, *GAD-7* Generalised Anxiety Disorder Screener-7, *PHQ-15* Patient Health Questionnaire-15, *EBQ* Emotion Beliefs Questionnaire, *ERQ* Emotion Regulation Questionnaire, *BEAQ* Brief Experiential Avoidance Questionnaire, *PID5BF+M* Modified Personality Inventory for DSM-5 Brief Form

Transdiagnostic factor scores generally followed a stepwise pattern aligned with the respective depressive, anxiety and somatic symptom severity levels. Clear differentiation between severity levels was limited as transdiagnostic score distributions overlapped across severity levels; some transdiagnostic factor scores also showed inconsistent patterns (e.g. beliefs about emotions across somatic symptom severity levels). The most consistent separation across all three symptom domain severity levels was observed for negative affectivity (Fig. 3).

### Discussion

This cross-sectional analysis explored how transdiagnostic factors, including emotion regulation mechanisms and negative affectivity, correspond to depressive, anxiety and somatic symptoms in PC, and assessed whether emotion regulation mechanisms have potential screening relevance beyond negative affectivity.

As implied by previous research [38], all transdiagnostic factors were correlated with diagnosis-specific symptom scores. Consistent with its proposed role as a general factor underlying internalising psychopathology in PC patients [14, 39], negative affectivity demonstrated the strongest associations across depressive, anxiety and somatic symptoms. By contrast, small to moderate correlations were indicated by emotion beliefs, cognitive reappraisal, emotion suppression and experiential avoidance, suggesting more limited potential relevance in screening contexts. Among these mechanisms, experiential avoidance showed the strongest associations, consistent with the notion that maladaptive regulation strategies may be more closely related to psychopathology than adaptive strategies [40]. Nevertheless, although emotion-regulation mechanisms are conceptually related to negative affectivity -as individuals high in negative affectivity tend to engage more frequently in maladaptive regulation strategies-they can be considered more proximal processes describing how negative emotions are managed instead of the general tendency to experience them [15]. Thus, while overlapping with negative affectivity, they may retain translational value for informing treatment planning by specifying modifiable targets [15, 17].

As opposed to previous findings [38], the emotion regulation mechanisms did not demonstrate potential screening relevance beyond negative affectivity. Only cognitive reappraisal implied some indication of additional explanatory value for depressive symptoms, an association that aligns with previous research [41]. In general, distinguishing the unique contribution of individual emotion regulation mechanisms remains challenging due to substantial conceptual overlap among these constructs [42]. This interrelatedness-compounded by the small sample size – likely contributed to instability in the regression coefficients, most apparent for emotion beliefs. Although point estimates for emotion beliefs were consistently negative across symptom domains, this pattern is most plausibly attributable to multicollinearity, as a genuine inverse association would not be expected [43].

With regard to the respective depressive, anxiety and somatic severity levels, negative affectivity demonstrated the most distinct gradient, thereby offering further support for its potential screening relevance in PC [14]. Across analyses, the stronger associations of negative affectivity with depressive, anxiety and somatic symptoms relative to the other transdiagnostic mechanisms, may partly reflect criterion overlap. Both negative affectivity and diagnosis-specific symptom measures assess negative emotional experiences [39], whereas emotion-regulation mechanisms capture how emotions are managed rather than their mere presence [15]. Accordingly, although a general trend of more maladaptive emotion regulation was indicated with increasing symptom severity, differentiation between specific severity levels remained limited. This may be linked to substantial inter-individual differences in emotion-regulation tendencies despite comparable symptom profiles; for example, two individuals with a similar symptom burden may nevertheless hold markedly different beliefs about their emotions [43].

Taken together, negative affectivity comparatively appeared to be a transdiagnostic factor with particular relevance for characterising overall mental health symptom burden in PC. In general, the relevance of emotion-regulation mechanisms for informing mechanism-based interventions and symptom change warrants further, prospective research.

## Limitations

Several limitations must be acknowledged. As the original pilot study was not designed or powered for the aims of this secondary analysis, the findings should be viewed as preliminary. The small sample size increases uncertainty around the derived estimates, further compounded by the multicollinearity among the transdiagnostic factors when considered simultaneously in multiple regression analyses. In addition, all outcomes were assessed exclusively through self-report measures, which may provide restricted insight into complex psychopathological constructs [40]. The limited internal consistency of the PHQ-15 (α = 0.60) may further constrain the robustness of findings related to somatic symptoms, as lower alpha values may reflect weaker item interrelatedness and greater construct heterogeneity [44]. This may partly correspond to the heterogeneous presentation of somatic symptoms in PC [45]. Also, diagnostic accuracy metrics were not evaluated in the present sample, including sensitivity, specificity or symptom severity cut-offs. Accordingly, and particularly given the cross-sectional design, the findings should not be interpreted as evidence of validated real-world screening utility, but rather as an exploratory examination of potential screening relevance. Future research should replicate these analyses with an adequately powered sample.

## Supplementary Information

Below is the link to the electronic supplementary material.

Additional file 1. Linear associations between transdiagnostic factor scores and depressive symptom score (PHQ-9).

Additional file 2. Linear associations between transdiagnostic factor scores and anxiety symptom score (GAD-7).

Additional file 3. Linear associations between transdiagnostic factor scores and somatic symptom score (PHQ-15).

Additional file 4. Multiple regression estimates of transdiagnostic factor scores on diagnosis-specific symptom scores.

## Data Availability

The dataset analysed for the current study, together with the statistical code, are available in the Open Science Framework (OSF) repository: 10.17605/OSF.IO/96U34.

## Abbreviations

BEAQ: Brief Experiential Avoidance Questionnaire
EBQ: Emotion Beliefs Questionnaire
ERQ: Emotion Regulation Questionnaire
GAD-7: Generalised Anxiety Disorder Screener-7
GP: General practitioners
PC: Primary care
PHQ-15: Patient Health Questionnaire-15
PHQ-9: Patient Health Questionnaire-9
PID5BF+M: Modified Personality Inventory for DSM-5 Brief Form
