# Supplementary figures and images for: Exploring transdiagnostic factors for mental health screening in primary care: a secondary analysis of a randomised controlled pilot study
Source: BMC Res Notes. 2026 May 29;19:239. doi: 10.1186/s13104-026-07885-5 (PMC13221761; doi:10.1186/s13104-026-07885-5)

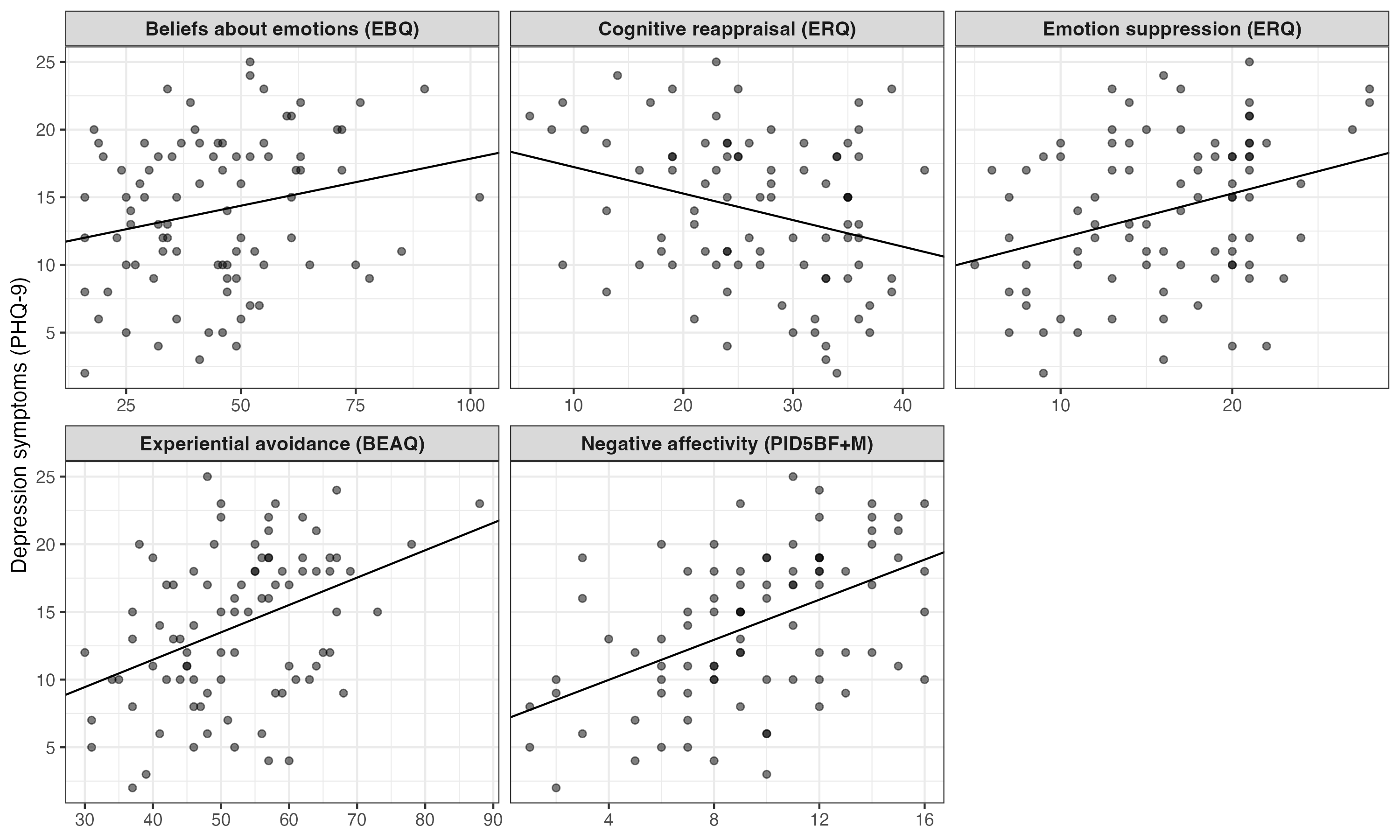

Supplement: Supplementary file 1 — Additional file 1. Linear associations between transdiagnostic factor scores and depressive symptom score (PHQ-9). [file 13104_2026_7885_MOESM1_ESM.png]

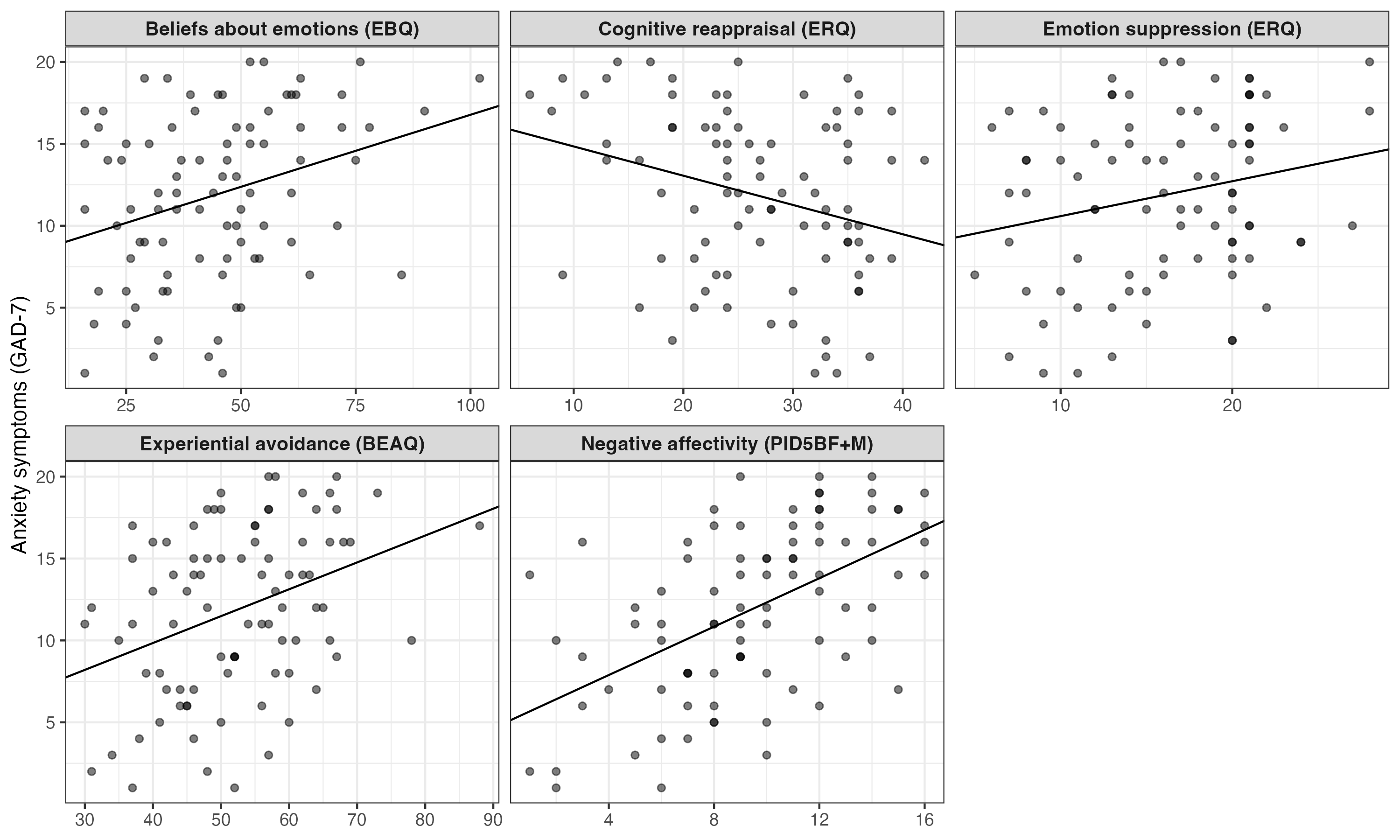

Supplement: Supplementary file 2 — Additional file 2. Linear associations between transdiagnostic factor scores and anxiety symptom score (GAD-7). [file 13104_2026_7885_MOESM2_ESM.png]

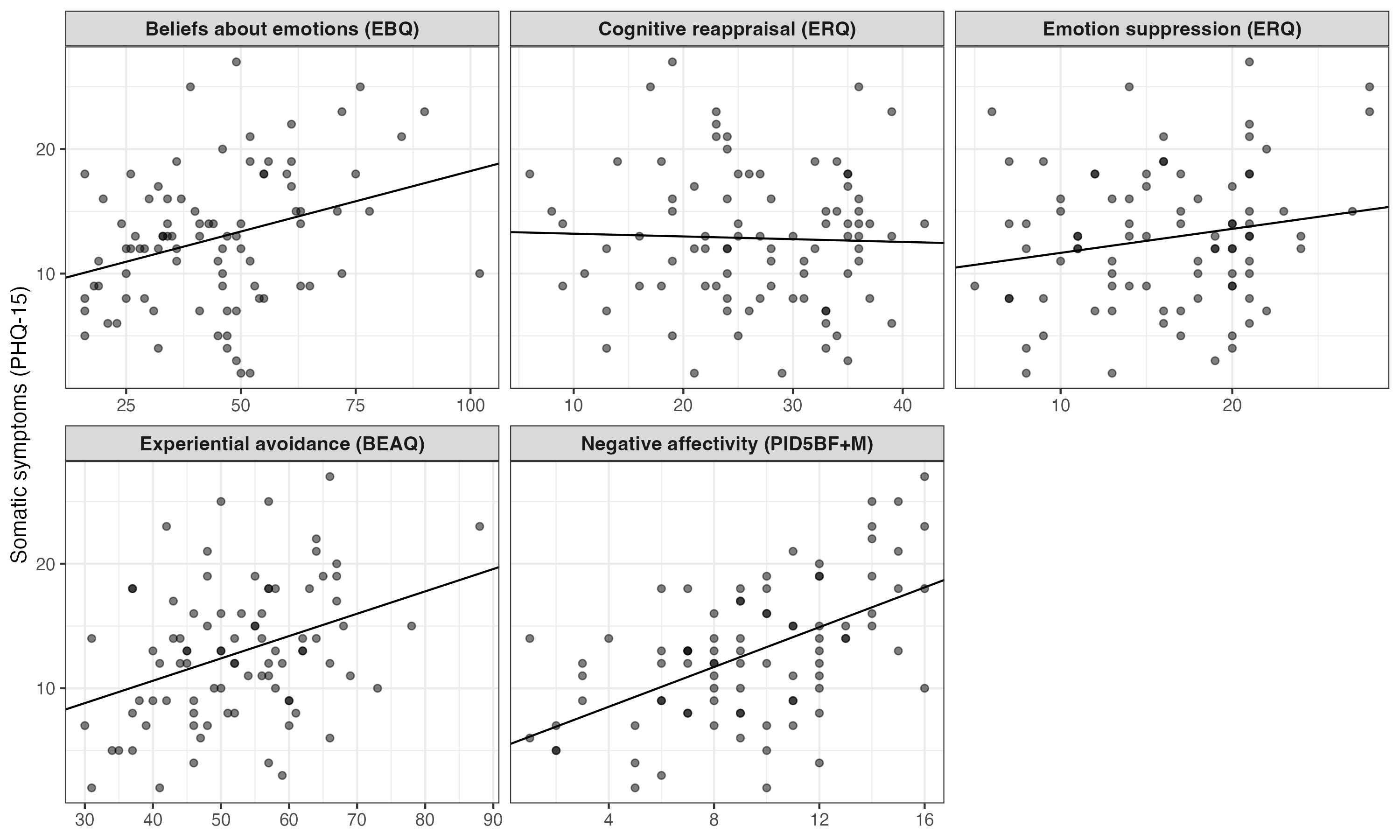

Supplement: Supplementary file 3 — Additional file 3. Linear associations between transdiagnostic factor scores and somatic symptom score (PHQ-15). [file 13104_2026_7885_MOESM3_ESM.png]
