# Supplementary material for: Exploring transdiagnostic factors for mental health screening in primary care: a secondary analysis of a randomised controlled pilot study
Source: BMC Res Notes. 2026 May 29;19:239. doi: 10.1186/s13104-026-07885-5 (PMC13221761; doi:10.1186/s13104-026-07885-5)
Supplement: Supplementary file 4 — Additional file 4. Multiple regression estimates of transdiagnostic factor scores on diagnosis-specific symptom scores. [file 13104_2026_7885_MOESM4_ESM.docx]

# Additional file 4. Multiple regression estimates of transdiagnostic factor scores on diagnosis-specific symptom scores

| Standardised regression estimates | | | | | | |
| --- | --- | --- | --- | --- | --- | --- |
|  | R^2^ | Beliefs about emotions (EBQ) | Cognitive  reappraisal (ERQ) | Emotion  suppression (ERQ) | Experiential avoidance (BEAQ) | Negative  affectivity (PID5BF+M) |
| VIF |  | 1.759 | 1.112 | 1.539 | 2.323 | 2.015 |
| Depressive symptoms (PHQ9) | .301 | -1.240  [-2.477, -.003] | -1.221*  [-2.294, -.147] | .913  [-.387, 2.213] | .981  [-.633, 2.596] | 2.387**  [.881, 3.893] |
| Anxiety  symptoms (GAD7) | .290 | -.383  [-1.715, .949] | -.924  [-1.894, .046] | .343  [-.939, 1.626] | .404  [-1.288, 2.095] | 2.430**  [.956, 3.895] |
| Somatic  symptoms (PHQ15) | .270 | -.196  [-1.843, 1.451] | .578  [-.462, 1.618] | .074  [-1.336, 1.485] | .268  [-1.599, 2.135] | 3.082***  [1.526, 4.638] |
